# Ceftolozane-Tazobactam for the Treatment of Multidrug-Resistant *Pseudomonas aeruginosa* Infections: A Multicenter Study

**DOI:** 10.1093/ofid/ofy280

**Published:** 2018-10-31

**Authors:** Jason C Gallagher, Michael J Satlin, Abdulrahman Elabor, Nidhi Saraiya, Erin K McCreary, Esther Molnar, Claudine El-Beyrouty, Bruce M Jones, Deepali Dixit, Emily L Heil, Kimberly C Claeys, Jon Hiles, Nikunj M Vyas, Christopher M Bland, Jin Suh, Kenneth Biason, Dorothy McCoy, Madeline A King, Lynette Richards, Nicole Harrington, Yi Guo, Saira Chaudhry, Xiaoning Lu, Daohai Yu

**Affiliations:** 1 Temple University, Department of Pharmacy Practice, Philadelphia, Pennsylvania; 2 Division of Infectious Diseases, Weill Cornell Medicine, New York, New York; 3 Division of Infectious Diseases, Temple University Hospital, Philadelphia, Pennsylvania; 4 Department of Pharmacy, Montefiore Medical Center, Wakefield Campus, Bronx, New York; 5 Department of Pharmacy, University of Pittsburgh Medical Center, Pennsylvania; 6 Department of Pharmacy, Thomas Jefferson University Hospital, Philadelphia, Pennsylvania; 7 Department of Pharmacy, St. Joseph’s/Candler Health System, Savannah, Georgia; 8 Department of Pharmacy Practice, Rutgers University, Ernest Mario School of Pharmacy, Pisacataway, New Jersey; 9 Department of Pharmacy Practice, University of Maryland School of Pharmacy, Baltimore; 10 Department of Pharmacy, Indiana University Health, Indianapolis; 11 Department of Pharmacy, Jefferson Health New Jersey, Stratford, New Jersey; 12 Department of Pharmacy Practice, University of Georgia College of Pharmacy, Savannah; 13 Division of Infectious Diseases, St. Joseph’s University Medical Center, Paterson, New Jersey; 14 Department of Pharmacy, St. Joseph’s University Medical Center, Paterson, New Jersey; 15 University of the Sciences, Philadelphia College of Pharmacy, Pennsylvania; 16 St. Joseph’s Wayne Medical Center, Wayne, New Jersey; 17 Department of Pharmacy, Christiana Care Health System, Newark, Delaware; 18 Department of Pharmacy, Montefiore Medical Center, Bronx, New York; 19 Department of Clinical Services, Temple University, Philadelphia, Pennsylvania

**Keywords:** ceftolozane-tazobactam, Gram-negative, multidrug-resistant infections, *Pseudomonas*, resistance

## Abstract

**Background:**

Multidrug-resistant *Pseudomonas aeruginosa* infections remain common in hospitals worldwide. We investigated the outcomes associated with the use of ceftolozane-tazobactam for the treatment of these infections.

**Methods:**

Data were collected retrospectively from 20 hospitals across the United States about adults who received ceftolozane-tazobactam for the treatment of multidrug-resistant *P aeruginosa* infections of any source for at least 24 hours. The primary outcome was a composite of 30-day and inpatient mortality, and secondary outcomes were clinical success and microbiological cure. Multivariable regression analysis was conducted to determine factors associated with outcomes.

**Results:**

Two-hundred five patients were included in the study. Severe illness and high degrees of comorbidity were common, with median Acute Physiology and Chronic Health Evaluation (APACHE) II scores of 19 (interquartile range [IQR], 11–24) and median Charlson Comorbidity Indexes of 4 (IQR, 3–6). Delayed initiation of ceftolozane-tazobactam was common with therapy started a median of 9 days after culture collection. Fifty-nine percent of patients had pneumonia. On susceptibility testing, 125 of 139 (89.9%) isolates were susceptible to ceftolozane-tazobactam. Mortality occurred in 39 patients (19%); clinical success and microbiological cure were 151 (73.7%) and 145 (70.7%), respectively. On multivariable regression analysis, starting ceftolozane-tazobactam within 4 days of culture collection was associated with survival (adjusted odds ratio [OR], 5.55; 95% confidence interval [CI], 2.14–14.40), clinical success (adjusted OR, 2.93; 95% CI, 1.40–6.10), and microbiological cure (adjusted OR, 2.59; 95% CI, 1.24–5.38).

**Conclusions:**

Ceftolozane-tazobactam appeared to be effective in the treatment of multidrug-resistant *P aeruginosa* infections, particularly when initiated early after the onset of infection.

Infectious diseases are forever evolving, but the threat of multidrug-resistant (MDR) *Pseudomonas aeruginosa* infections is constant. These difficult-to-treat organisms account for a significant percentage of hospital-acquired infections, and options to treat them are limited. Unlike carbapenem-resistant *Enterobacteriaceae* infections, MDR *P aeruginosa* isolates are usually susceptible to several antimicrobial agents [1]. The most consistently active drugs upon susceptibility testing are aminoglycosides and polymyxins, agents that are suboptimal for the treatment of pseudomonal infections due to pharmacokinetic limitations and their association with worse outcomes when given as monotherapy [2, 3].

Ceftolozane-tazobactam is a combination agent that consists of a novel antipseudomonal cephalosporin combined with a β-lactamase inhibitor in a 2:1 ratio [4, 5]. Ceftolozane has improved activity against *P aeruginosa* relative to most β-lactams because it is stable against AmpC enzymes produced by this organism, is not affected by active efflux, and is not appreciably affected by porin channel changes. Tazobactam protects ceftolozane from destruction by most extended-spectrum β-lactamases but does not add to its activity against *P aeruginosa* [4, 5]. At present, the combination is indicated for the treatment of complicated urinary tract infections and complicated intra-abdominal infections, the latter with concomitant metronidazole. Despite potent in vitro activity against strains of *P aeruginosa* that are resistant to other antipseudomonal β-lactam agents, few of these isolates were in clinical trials, and clinical evidence supporting ceftolozane-tazobactam for these infections is lacking. To investigate this, we conducted a multicenter retrospective study.

## METHODS

This was a multicenter, retrospective observational study of patients who were treated with ceftolozane-tazobactam for MDR *P aeruginosa* infections from any source between December 2014 and February 2018 at 20 health systems in the United States. To be included, isolates had to be resistant to at least 1 antipseudomonal agent in 3 drug classes, in agreement with published criteria [6]. Patients ≥18 years old who received at least 24 hours of ceftolozane-tazobactam therapy were included. Prisoners, pregnant women, and children were excluded. Patients were identified through electronic medical records, and patient information was entered into a REDcap database [7]. The study was approved by the institutional review boards of each participating site.

Data on baseline, infection, and demographic characteristics were assessed from the time of the index infection. We used US Centers for Disease Control and Prevention criteria to define infections as assessed by individual investigators and reviewed by primary investigators (J.C.G. and E.M.) [8]. The degree of comorbid illness was assessed using the Charlson comorbidity index (CCI), and Acute Physiology and Chronic Health Evaluation (APACHE) II scores were calculated to assess the severity of illness [9, 10]. Clinical characteristics were evaluated on the day that ceftolozane-tazobactam was started. Dosing of ceftolozane-tazobactam was determined by providers at each institution. For the purposes of categorization, patients receiving 3 gm IV q8h or the renally adjusted equivalent were considered to receive high-dose ceftolozane-tazobactam, and patients who were treated via recommended doses in the package insert were considered to have normal dosing [11]. To analyze dosing strategies, creatinine clearance (CrCl) was calculated by the lead site. Concomitant intravenous antibiotic therapy and prior therapy for pseudomonal infections were recorded. Determinations of susceptibility were made at each site using US Food and Drug Administration (FDA) breakpoints for ceftolozane-tazobactam and Clinical and Laboratory Standards Institute (CLSI) breakpoints for other drugs [4, 12]. Due to the lack of an FDA-approved susceptibility test at the initiation of the study, testing for ceftolozane-tazobactam susceptibility was not required for enrollment, although susceptibility results were collected when available.

The primary outcome was a composite of 30-day and inpatient all-cause mortality, where patients who were discharged before 30 days were considered survivors without other information, and those who died in the hospital after 30 days were also considered survivors. Patients who were discharged before 30 days, readmitted, and died within 30 days of the infection were included as negative outcomes. Secondary outcomes were microbiologic cure, defined as a negative culture at the end of therapy, and clinical success, defined as improved signs and symptoms from baseline to the end of therapy with defervescence. Microbiologic cure was presumed in surviving patients with clinical success when repeat cultures were not available. Outcomes were assessed by site investigators and adjudicated by 2 of the investigators (J.C.G. and E.M.).

Descriptive summary data were expressed as counts and percentages for categorical variables and medians (interquartile range [IQR]) for continuous variables. Comparisons between groups were performed using the Fisher’s exact or χ^2^ test for categorical variables and the Wald test for continuous variables via univariable logistic regression of outcomes. Associations between outcomes and potential risk factors were first evaluated using raw/unadjusted odds ratios (ORs) and 95% confidence intervals (CIs). Ordinal or continuous covariates were also considered in the analysis in a categorized version using their medians and/or clinical meaningful cut points (eg, delay in therapy of 5 days or more vs 4 days or under). Furthermore, multivariable logistic regression models were used to examine the associations of individual predictor variables with the primary or secondary outcome variables one at a time while adjusting for all potential confounding factors or effect-modifiers. Variable selection was implemented in a stepwise fashion. Adjusted ORs and their 95% CIs based on these regression models are reported as appropriate. No multiple comparison adjustments were made. Two-tailed *P* values of less than 0.05 were considered statistically significant. SAS version 9.4 (SAS Institute, Cary, NC) was used to carry out all the data analyses.

## RESULTS

Patient characteristics are in Table 1. Two hundred five patients were included in the study. Notable characteristics include prolonged hospital durations with a median length of stay of 31.5 days (IQR, 14.5–65.0), significant degrees of comorbid illness with a median CCI of 4 (IQR, 3–6), and severe illness with median APACHE II scores of 19 (IQR, 11–24). Delays from when *Pseudomonas* cultures were collected to when ceftolozane-tazobactam was started were common, with a median delay of 9 days. Notable comorbidities included organ transplantation in 35 patients (17.1%), pulmonary disease in 82 (40.0%), diabetes in 69 (33.7%), and cancer in 33 (16.1%).

**Table 1.** Patient Characteristics (n = 205)

Patient Characteristics	Results
Age, median (IQR)	60 (48–70)
Weight (kg), median (IQR)	74.5 (64.0–90.5)
LOS, median (IQR)	31.5 (14.5–65.0)
Male gender, n (%)	120 (58.5)
Charlson Comorbidity Index, median (IQR)	4 (3–6)
Comorbidities, n (%)	
Solid organ transplant	35 (17.1)
Pulmonary disease	82 (40.0)
Diabetes mellitus	69 (33.7)
Heart failure	47 (22.9)
Renal disease	54 (26.3)
Liver disease	22 (10.7)
Cancer	33 (16.1)
APACHE II score, median (IQR)	19 (11–24)
ICU at time of infection, n (%)	105 (51.2)
Therapy Characteristics	Results
Hospital day infection isolated, median (IQR)	2 (1–21)
Hospital day ceftolozane-tazobactam started, median (IQR)	11 (4–30)
Concomitant antipseudomonal antibiotics given, n (%)	81 (39.5)
Duration of ceftolozane-tazobactam therapy, median (IQR)	10 (7–14)
High-dose therapy^a^, n (%)	97 (47.3)
Renally adjusted dose^b^, n (%)	63 (30.7)

Abbreviations: APACHE, Acute Physiology and Chronic Health Evaluation; CrCl, creatinine clearance; ICU, intensive care unit; IQR, interquartile range; LOS, length of stay.

^a^3 gm IV q8h in patients with CrCl >50 mL/min; patients with CrCl <50 mL/min were considered to receive high-dose therapy when it was above the recommendations in the package insert.

^b^Adjusted for renal function by the package insert or as noted as high-dose therapy.

Patients with many different types of infection were included. Pneumonia was the most common, occurring in over half of patients (121 [59.0%]), followed by infections of the urinary tract, wounds, abdomen, bone/joint, and bloodstream. In addition to the 6 patients with bloodstream infections with no other source identified, 19 patients had secondary bacteremia with a known source. Table 2 describes the resistance patterns of the cultured pathogens. All patients had MDR *P aeruginosa* infections, which were most commonly nonsusceptible to antipseudomonal carbapenems (96.8%), piperacillin-tazobactam (94.2%), aztreonam (92.8%), and cefepime/ceftazidime (85.6%). Over 70% of pathogens were susceptible to tobramycin and amikacin, but only 48% were gentamicin-susceptible. Ceftolozane-tazobactam susceptibility was tested in 139 (67.8%) of patients and was susceptible in 125 cases (89.9%).

**Table 2.** Isolate Susceptibilities^a^

Drug tested, %Susceptible (n/N Tested)							
AMK	ATM	FEP/CAZ	GEN	MEM/IPM	TZP	TOB	CST
78.6% (125 of 159)	7.2% (10 of 139)	14.4% (21 of 177)	57.7% (94 of 163)	3.4% (6 of 179)	5.8% (10 of 173)	74.3% (104 of 140)	91.9% (57 of 62)
Ceftolozane-tazobactam (n/N tested, % susceptible)				125 of 139 (89.9%)			

Abbreviations: AMK, amikacin; ATM, aztreonam; CAZ, ceftazidime; CST, colistin; FEP, cefepime; GEN, gentamicin; IPM, imipenem; MEM, meropenem; TOB, tobramycin; TZP, piperacillin-tazobactam.

^a^Most centers reported results for either FEP or CAZ and MEM or IPM. Results here reflect the combined data for both. Not all centers tested all listed drugs.

Outcomes are described in Table 3. Overall, 30-day or inpatient mortality occurred in 39 patients (19.0%). Clinical success was seen in 151 (73.7%) patients, and microbiological cure occurred in 145 (70.7%) patients. Success rates for all endpoints were lowest in pneumonia, particularly for microbiological cure, where repeated cultures were most common. On multivariable regression analysis, pneumonia was a strong predictor of microbiological failure (adjusted OR, 8.06; 95% CI, 3.34–19.49), but not clinical failure or mortality.

**Table 3.** Clinical Outcome Data Summary

Overall Outcomes		Results, n (%)	
Mortality		39 (19.0)	
Clinical success		151 (73.7)	
Microbiological cure		145 (70.7)	
Outcomes by infection type			
Type (n)^a^	Microbiological Cure, n (%)	Clinical Success, n (%)	Mortality, n (%)
Bloodstream, primary^b^ (6)	6 (100)	6 (100)	0 (0)
Bone/joint (16)	13 (81.3)	13 (81.3)	0 (0)
Intra-abdominal (20)	18 (90.0)	15 (75.0)	2 (10.0)
Pneumonia (121)	69 (57.0)	80 (66.1)	31 (25.6)
VAP (58)	31 (53.4)	29 (50.0)	22 (37.9)
Non-VAP (63)	38 (60.3)	51 (81.0)	9 (14.2)
Wound (26)	21 (80.8)	21 (80.8)	4 (15.4)
UTI (28)	25 (89.3)	25 (89.3)	4 (14.3)

Abbreviations: UTI, urinary tract infection; VAP, ventilator-associated pneumonia.

^a^Some patients had *Pseudomonas* isolated from multiple sites with multiple types of infection diagnosed; therefore, the total is greater than 205.

^b^Positive blood cultures for *Pseudomonas* with no other identified source of infection. In addition, 19 patients had secondary bacteremia. Of these 19, 13 (68%) had both microbiological cure and clinical success, and 7 (36.8%) died.

Results of the multivariable analysis are available in Table 4. Most notably, initiation of ceftolozane-tazobactam within 4 days of the culture collection was a significant predictor of survival, clinical success, and microbiological success. This relationship was also significant when analyzing the data for initiation of ceftolozane-tazobactam within 5 days and at 3 days postdiagnosis (data not shown). It is notable that high-dose ceftolozane-tazobactam and concomitant antibiotics were not associated with positive outcomes, although concomitant antibiotics were associated with microbiological failure (adjusted OR, 3.85; 95% CI, 1.86–7.93). Age ≥60 had an unusual protective effect for 30-day mortality, although the median age for those with a positive outcome (ie, microbiological cure) was only 5 years apart from those with a negative outcome (62 vs 57 years old).

**Table 4.** Multivariable Analysis of Factors Associated With Clinical Outcomes

Effect	Point Estimate of Odds Ratio (OR)	95% Confidence Interval for OR
Mortality		
Ceftolozane-tazobactam started >4 days after culture	5.55	2.14–14.40
Age ≥60	0.20	0.07–0.57
Charlson Comorbidity Index (each 1 point)	1.24	1.01–1.52
Vasopressor use	5.68	2.15–14.98
APACHE II score (each 1 point)	1.14	1.08–1.22
Clinical success		
Ceftolozane-tazobactam started ≤4 days after culture	2.93	1.40–6.10
Vasopressor use	0.16	0.070–0.344
APACHE II score (each 1 point)	0.95	0.91–0.99
Microbiological cure		
Ceftolozane-tazobactam started ≤4 days after culture	2.59	1.24–5.38
Concomitant antibiotic use	0.26	0.13–0.54
Pneumonia	0.12	0.05–0.30
Vasopressor use	0.33	0.15–0.73

Abbreviations: APACHE, Acute Physiology and Chronic Health Evaluation; OR, odds ratio.

## DISCUSSION

To our knowledge, this is the largest study to date evaluating ceftolozane-tazobactam for MDR *P aeruginosa* infections. The population included was largely critically ill, demonstrated by high APACHE II scores and CCIs, the need for renally adjusted dosing, and intensive care unit admission. As one may expect for a then newly introduced antipseudomonal agent, delays in therapy were common. It is important to examine the results of the study in the context of the degree of illness of the patients included. The overall mortality rate of 19% is comparable with other studies of patients with invasive *P aeruginosa* infections [13–19]. Several studies have found that both multidrug resistance and delays in appropriate therapy increase mortality in *Pseudomonas* infections, particularly for bloodstream infections [15–19]. In our study, all patients had MDR infections, and increased time to ceftolozane-tazobactam initiation was significantly associated with mortality.

It is interesting that no positive effects were observed through the use of concomitant intravenous antibiotics or high-dose ceftolozane-tazobactam, even though these practices were common. In fact, concomitant antibiotics were associated with microbiological failure, likely due to selection bias wherein patients who were doing poorly had clinicians who elected to administer additional antibiotics. The lack of a detected positive effect from concomitant therapy suggests that ceftolozane-tazobactam monotherapy may be sufficient for the treatment of *P aeruginosa* infections that are susceptible to this agent.

As expected, pneumonia represented the majority of included infections, and, unsurprisingly, this also had the worst outcomes because these patients were generally on ventilators with multiple comorbidities. The difference between microbiological cure (57%) and clinical success (66.1%) in these patients may be due to repeated respiratory samples growing *P aeruginosa* in ventilated patients who clinically improved, given that the observed 71.9% survival was closer to clinical than microbiological results.

The finding that earlier initiation of ceftolozane-tazobactam after culture collection was associated with lower mortality, greater clinical success, and greater microbiological cure is significant. Although de-escalation approaches are commonly recommended by clinical guidelines in many syndromes, the elevated costs associated with new antimicrobials agents and a desire to reserve these agents for highly resistant organisms may lead to a reversion to an “escalation” approach in patients with these organisms. The fact that the median time from culture collection to ceftolozane-tazobactam initiation in our study was 9 days is evidence of this. Antimicrobial stewardship programs and individual practitioners alike need to strike a difficult balance between early use of agents such as ceftolozane-tazobactam in appropriate patients who may benefit from it and the economic and microbiological consequences of overuse. The increased use of rapid diagnostics and scoring systems for MDR organisms may be of use to identify patients who could benefit from earlier initiation of ceftolozane-tazobactam therapy, although the ability to rapidly differentiate MDR from susceptible phenotypes is limited.

This study examined outcomes of patients treated with ceftolozane-tazobactam for MDR *P aeruginosa* infections in multiple hospitals across the United States. Published reports in the literature for this indication are limited. A group at the Cleveland Clinic Health System summarized the use of ceftolozane-tazobactam in their system [20]. Although 86.7% of the 60 included cases had *P aeruginosa* infections, only 40.4% of those were MDR, and results were not reported specifically for that subset. Only 10% of patients in that study received high-dose ceftolozane-tazobactam compared with 47.3% of our patients, which may represent either evolving practices or differences between institutions. Two reports have described ceftolozane-tazobactam specifically for phenotypically MDR infections [21, 22]. In a study of patients in 6 hospitals, Munita et al [22] described clinical success in 26 of 35 patients (74%). Haidar et al [21] evaluated 21 patients at the University of Pittsburgh Medical Center and reported that 19 of 21 (90%) survived to 30 days. We await the publication of the recently completed ASPECT-NP study (NCT02070757) of ceftolozane-tazobactam for ventilated hospital-acquired pneumonia for more evidence supporting its use for pseudomonal infections.

## CONCLUSIONS

Our study is limited by its retrospective nature and lack of a control group to evaluate comparative outcomes. The real-world nature and the inclusion of 20 US medical centers are strengths of the study because it reflects observed practices throughout the country, but it has the weakness of its observational nature and inability to determine whether characteristics associated with outcomes are causative or circumstantial. However, we believe the study supports microbiological, pharmacokinetic, and early clinical evidence that ceftolozane-tazobactam is effective for infections caused by MDR *P aeruginosa*, including in critically ill patients.
